# Efficacy and safety of thrombolysis for acute ischemic stroke with atrial fibrillation: a meta-analysis

**DOI:** 10.1186/s12883-021-02095-x

**Published:** 2021-02-11

**Authors:** Yunzhen Hu, Chunmei Ji

**Affiliations:** 1 Department of Pharmacy, The First Affiliated Hospital, College of Medicine, Zhejiang University, Hangzhou, China; 2 Research Division of Clinical Pharmacology, the First Affiliated Hospital of Nanjing Medical University, Nanjing, Jiangsu China

**Keywords:** Meta-analysis, Atrial fibrillation, Ischemic stroke, Thrombolysis

## Abstract

**Background:**

The efficacy and safety of intravenous thrombolysis (IVT) for acute ischemic stroke with atrial fibrillation (AF) is still controversial.

**Methods:**

We conducted a meta-analysis of all relevant studies, retrieved through systematic search of PubMed, Embase, and Cochrane databases up to December 31, 2019. Modified Rankin Scale (mRS) scores of 0–1 at 90 days, mRS of 0–2 at 90 days, overall mortality, and incidence of symptomatic intracranial hemorrhage (sICH) were collected as outcome measures. Fixed- and random-effects meta-analytical models were applied, and between-study heterogeneity was assessed.

**Results:**

A total of 8509 patients were enrolled in 18 studies. A comparison of IVT treatment in AF versus non-AF patients showed that AF was associated with a significantly lower proportion of patients with mRS of 0–1 (24.1% vs. 34.5%; OR 0.59; 95% CI 0.43–0.81; *P* < 0.001), mRS of 0–2 (33.6% vs. 47.8%; OR 0.55; 95% CI 0.43–0.70; *P* < 0.001), as well as significantly higher mortality (19.4% vs. 11.5%; OR 2.05; 95% CI 1.79–2.36; *P* < 0.001) and higher incidence of sICH (6.4% vs. 4.1%; OR 1.60; 95% CI 1.27–2.01; *P* < 0.001). A comparison of AF patients who were subjected or not to IVT showed that thrombolysis carried a higher risk of sICH (5.7% vs. 1.6%; OR 3.44; 95% CI 2.04–5.82; *P* < 0.001) and was not associated with a better prognosis. Subgroup analysis in prospective studies also suggested a poorer functional prognosis and higher mortality in AF patients treated with IVT compared with those who did not receive IVT. Some heterogeneity was present in this meta-analysis.

**Conclusions:**

Acute IS patients with AF had worse outcomes than those without AF after thrombolytic therapy, and had a higher incidence of sICH after thrombolysis than those without thrombolysis. Thrombolysis in ischemic stroke patients with AF should be carefully considered based on clinical factors such as NIHSS score, age, and the type of AF.

## Background

Atrial fibrillation (AF) is a major risk factor for cardioembolic stroke, which is responsible for up to one-third of all ischemic stroke (IS) cases [1]. AF is associated with a 4–5-fold increased risk of IS [2], and AF-related strokes are more frequently fatal or disabling than those without a history of AF [3]. Intravenous thrombolysis (IVT) in acute IS patients within 4.5 h of onset can significantly improve functional outcome and reduce the risk of death and severe disability from stroke [4].

What is the efficacy and safety of thrombolysis for acute IS with AF? There are different opinions on this subject. When IS patients with and without AF are compared, most studies indicate that patients without AF show better 90-day functional outcomes after receiving thrombolytic therapy than those with AF [5–13]. However, a few studies reach the opposite conclusion [14, 15]. In addition, most studies show that AF patients pretreated with IVT have higher mortality [5–12, 15–19] and sICH incidence [5, 7–12, 16, 17, 19] than those without AF. When comparing AF patients treated or not with IVT, some studies indicate that IVT is associated with much better functional outcomes [7, 9, 15, 20, 21]. However, several other studies reach the opposite conclusion [5, 11]. In the case of AF patients treated with IVT therapy, five studies showed lower mortality [7, 9, 15, 20, 21] with respect to those who did not receive IVT therapy. On the contrary, two studies reported higher mortality [5, 11]. Due to these controversial results, we performed a meta-analysis of all relevant studies measuring the efficacy and safety of thrombolytic therapy for acute IS patients with AF.

## Methods

### Data sources and search strategy

We used PubMed, Embase and Cochrane electronic databases to identify all published studies assessing the efficacy and safety of thrombolytic therapy for acute IS with AF, up to December 31, 2019. The search terms were ((stroke) OR (cerebrovascular disorders) OR (cerebral infarction) OR (brain infarction)) AND ((tissue plasminogen activator) OR (alteplase) OR (thrombolytic therapy) OR (thrombolysis)) AND ((atrial fibrillation) OR (AFib) OR (AF)). The search did not have any language restrictions.

### Study selection

Two investigators (YZ Hu and CM Ji) independently performed the study selection. Studies were considered to be potentially eligible for this meta-analysis if they met the following criteria: (1) they compared the efficacy and safety of thrombolysis in AF versus non-AF patients; (2) they measured the efficacy and safety of IVT in AF patients and compared the outcome with AF patients not treated with IVT; (3) they included sufficient data on the modified Rankin Scale (mRS) 0–1 and mRS 0–2, mortality and the incidence of symptomatic intracranial hemorrhage (sICH). Exclusion criteria were as follows: (1) non-clinical studies, such as reviews, meta-analysis, case reports, letters, or comments; (2) no clinical outcome data.

### Quality assessment

The quality of the cohort studies was assessed using the Newcastle-Ottawa Quality Assessment Scale (NOS) [22]. This scale is recommended by the Cochrane Non-Randomized Studies Methods Working Group and consists of eight items that assess patient selection, study comparability and outcome. Studies with scores 0–3 are considered of low quality, 4–6 of moderate quality, and 7–9 of high quality. The RCT study was assessed with the Cochrane collaboration tool for assessing risk of bias.

The potential sources of heterogeneity explored by the mete-regression analysis. The studies’ characteristics were grouped as follows: the type of study design (prospective/ retrospective); stroke severity (NIHSS<16/≥16), thrombolysis time window (<3 h/≥3 h) and the different online year of study. Meta-regression with random effects model was preferred with aggregate-level data.

### Data extraction and outcome measures

Two investigators (YZ Hu and CM Ji) independently extracted data from the studies (authors, year of publication, design), population characteristics (number of patients, average age, sex ratio, presence of hypertension, diabetes mellitus and dyslipidemia, median baseline NIHSS score, onset to needle, mortality and incidence of sICH).

The primary efficacy endpoint was “excellent outcome” (mRS of 0–1, 90 days after stroke), and the secondary efficacy endpoint was “good outcome” (mRS of 0–2, 90 days after stroke). The primary safety endpoint was mortality and the secondary safety endpoint was sICH incidence.

### Statistical analysis

The proportion of patients with mRS of 0–1 and 0–2, the mortality and the sICH incidence were compared between AF IVT and non-AF IVT groups and (or) AF IVT versus AF non-IVT groups. We calculated the odds ratios (ORs) and corresponding 95% confidence intervals (CIs) for each outcome. The heterogeneity of the studies included in our article was assessed by means of the *I*^2^ test [23]. The *I*^2^ ranged from 0 to 100%. *I*^2^ > 50% indicated high heterogeneity and in these cases the random-effects model was used for meta-analysis. Otherwise, the fixed-effects model was used. A funnel plot and Egger’s test were used to assess publication bias in the meta-analysis. *P* < 0.05 was considered statistically significant. All statistical tests were performed using STATA software (11.0; StataCorp, College Station, TX, USA).

## Results

### Study characteristics

**Fig. 1:**
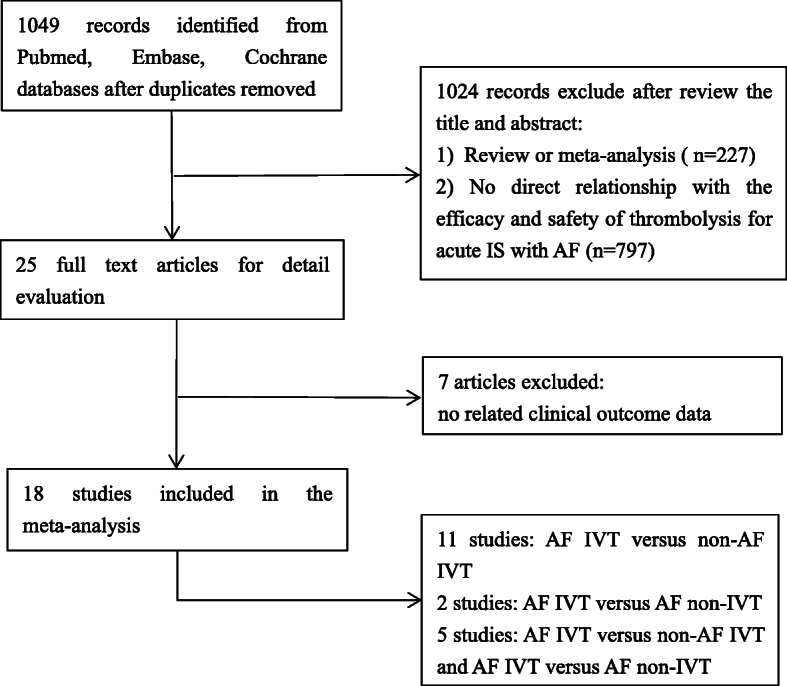
Literature search profile

We identified 1049 potentially relevant studies, but 1024 were excluded after screening the title and abstract. The full texts of the remaining 25 studies were retrieved for detailed evaluation. Based on the search criteria, a total of 18 studies reporting on the efficacy and safety of thrombolysis for acute IS with AF were included in this study (Fig. 1). The baseline characteristics of the patients included in these studies are shown in Tables 1 and 2. Eleven studies [6, 8, 10, 12–14, 16–19, 24] compared IVT treatment in AF versus non-AF patients, two studies [20, 21] compared AF patients treated or not with IVT, and five studies [5, 7, 9, 11, 15] included both comparisons.

**Table 1:** Characteristics of studies comparing AF IVT with non-AF IVT

Study	Study design	Comparison	No. of patients	Mean age, y	Men (%)	Treatment	Hypertension (%)	Diabetes mellitus (%)	Dyslipidemia (%)	Baseline NIHSS	Onset to needle (min)	Mortality, %	SICH, %
Bluhmki 2009 [5]	prospective RCT	AF/non-AF	53/365	NR	NR	rtPA 3–4.5 h	NR	NR	NR	NR	NR	22.6/5.5, 90d	17.0/6.6
Kimura 2009 [6]	prospective	AF/non-AF	44/41	77.2/69.4	61.4/70.7	rtPA < 3 h	63.6/51.2	18.2/22.0	13.6/31.7	17.3/12.3	148.3/147.6	18.2/7.3, 90d	NR
Awadh 2010 [24]	retrospective	AF/non-AF	74/154	76/66.4	40/59	rtPA < 4.5 h	70/64	16.2/7.8	19/22	14/ 14.5	162.7/173.3	NR	4.0/4.5
Sanak 2010 [16]	retrospective	AF/non-AF	66/91	68.1/66.5	57.6/65.9	rtPA < 3 h	NR	NR	NR	13.0/10.0	146.3/145.5	18.2/3.3, 7d	4.5/0
Zhang 2010 [7]	retrospective	AF/non-AF	22/31	68.3/60.7	40.9/74.2	rtPA < 4.5 h	72.7/38.5	13.6/6.5	NR	12.0/9.1	203.7/196.7	18.2/9.7, 90d	18.2/6.5
Seet 2011 [8]	retrospective	AF/non-AF	76/138	78.9 /71.5	42.1/53.6	rtPA < 3 h	78.9/75.4	10.5/14.5	50.0/51.4	13 /12	138.3/143.5	22.4/15.2, 90d	13.2/5.1
Frank 2012 [9]	retrospective	AF/non-AF	639/2388	74.2 /65.7	47.3/58.2	rtPA < 3 h	71.1/61.6	NR	NR	15 /13	NR	21.8/13.6, 90d	2.7/1.7
Padjen 2013 [10]	prospective	AF/non-AF	155/579	76 /64	41.9/55.6	rtPA	80.0/62.3	20.0/15.7	45.2/45.9	14/10	148/153	21.9/9.0, 90d	5.8/5.5
Saposnik 2013 [11]	prospective	AF/non-AF	316/1373	NR	NR	rtPA	NR	NR	NR	NR	NR	26.3/14.2, 30d	9.2/6.4
Sung 2013 [14]	retrospective	AF/non-AF	72/71	68.3/64.6	58.3/64.8	rtPA < 3 h	79.2/77.5	27.8/39.4	51.4/64.8	17.7/14.4	113/119	6.9/12.7, 90d	8.3/9.8
Al-Khaled 2014 [17]	prospective	AF/non-AF	387/620	NR	NR	rtPA < 4.5 h	NR	NR	NR	NR	up to 4.5 h	10.8/6.6, in-hospital	7.5/4.7
Saarinen 2014 [18]	retrospective	AF/non-AF	92/179	77 /69	46/55	NR < 3 h	74/63	32/17	NR	NR	NR	20/6, 90d	NR
Tu 2015 [19]	prospective	AF/non-AF	28/111	76.5/73	61/52	rtPA 3-6 h	64/64	21/26	29/32	16/11	NR	36/16, 90d	7.1/4.5
Zhao 2017 [12]	retrospective	AF/non-AF	30/93	69.8/63.5	46.7/67.7	rtPA < 4.5 h	73.3/65.6	30.0/18.3	30.0/39.8	12/8	227/174	13.3/2.2	13.3/5.4
Mehrpour 2019 [13]	retrospective	AF/non-AF	24/94	NR	NR	rtPA < 4.5 h	NR	NR	NR	NR	NR	NR	NR
Yang 2019 [15]	retrospective	AF/non-AF	47/56	71.2 /60.4	34.0/80.4	rtPA 3-9 h	74.5/64.3	19.1/23.2	NR	11.5 /9.3	322.9/370.4	10.6/7.1, 90d	2.1/3.6

**Table 2:** Characteristics of studies comparing IVT with non-IVT in AF pattents

Study	Study design	Comparison	No. of patients	age, y	Men (%)	Treatment	Hypertension (%)	Diabetes mellitus (%)	Dyslipidemia (%)	Baseline NIHSS	Onset to needle (min)	Mortality, %	SICH, %
Bluhmki 2009 [5]	prospective RCT	IVT/Non-IVT	53/55	NR	NR	rtPA 3–4.5 h	NR	NR	NR	NR	NR	22.6/14.5, 90d	17.0/6.6
Zhang 2010 [7]	retrospective	IVT/Non-IVT	22/44	68.3/70.4	40.9/43.2	rtPA < 4.5 h	72.7/63.6	13.6/20.5	NR	12.0/12.6	203.7/NR	18.2/20.5, 90d	18.2/6.8
Frank 2012 [9]	retrospective	IVT/Non-IVT	639/992	74.2/73.9	47.3/47.6	rtPA < 3 h	71.1/75.5	NR	NR	15 /14	NR	21.8/23.2, 90d	2.7/0.9
Saposnik 2013 [11]	prospective	IVT/Non-IVT	316/1373	NR	NR	rtPA	NR	NR	NR	NR	NR	26.3/18.1, 30d	NR
Padjen 2014 [20]	prospective	IVT/Non-IVT	34/97	68/72	58.8/51.5	rtPA < 4.5 h	94.1/87.6	20.6/24.7	29.4/38.8	11/14	NR	14.7/45.4, 90d	5.9/4.1
Zhao 2016 [21]	retrospective	IVT/Non-IVT	151/116	71.3/73.5	43.7/43.1	rtPA < 4.5 h	73.5/67.2	15.9/19.0	17.2/12.1	15.1/13.9	165.7/179.3	23.2/25.0, 90d	13.9/1.7
Yang 2019 [15]	retrospective	IVT/Non-IVT	47/31	71.2/74.7	34.0/58.1	rtPA 3-9 h	74.5/93.4	19.1/32.2	NR	11.5 /13.7	322.9 /310.4	10.6/14.8, 90d	2.1/0

### Outcome of thrombolysis in AF versus non-AF patients

**Fig. 2:**
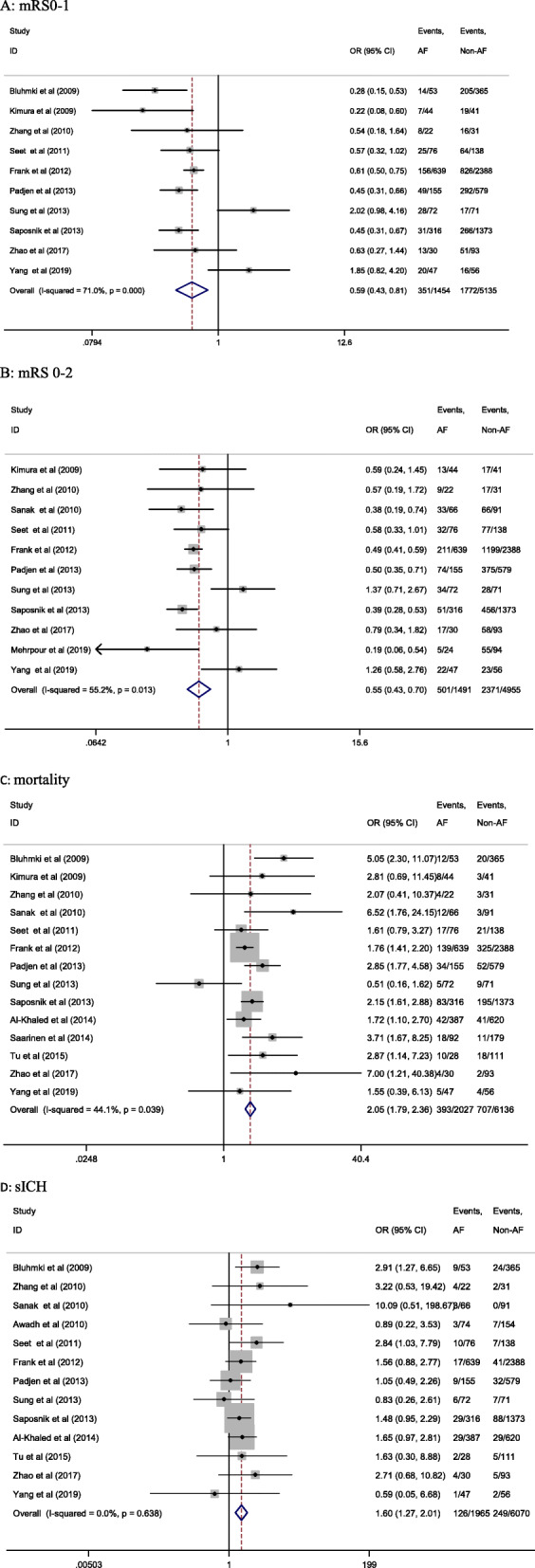
Forrest plot of meta-analysis of IVT in AF and non-AF patients. Each study is represented by a point estimate of the OR and the accompanying 95% CIs

In patients receiving thrombolytic therapy, significantly lower proportions of mRS 0–1 (24.1% vs. 34.5%; OR 0.59; 95% CI 0.43–0.81; *I*^2^ = 71.0%; *P* < 0.001, Fig. 2a) and mRS 0–2 (33.6% vs. 47.8%; OR 0.55; 95% CI 0.43–0.70; *I*^2^ = 55.2%; *P* < 0.001, Fig. 2b) scores were seen in patients with AF when compared with patients without AF. On the contrary, significantly higher mortality (19.4% vs. 11.5%; OR 2.05; 95% CI 1.79–2.36; *I*^2^ = 44.1%; *P* < 0.001, Fig. 2c) and sICH incidence (6.4% vs. 4.1%; OR 1.60; 95% CI 1.27–2.01; *I*^2^ = 0.0%; *P* < 0.001, Fig. 2d) were seen in AF patients.

### Comparing the outcome of AF patients treated or not with IVT

**Fig. 3:**
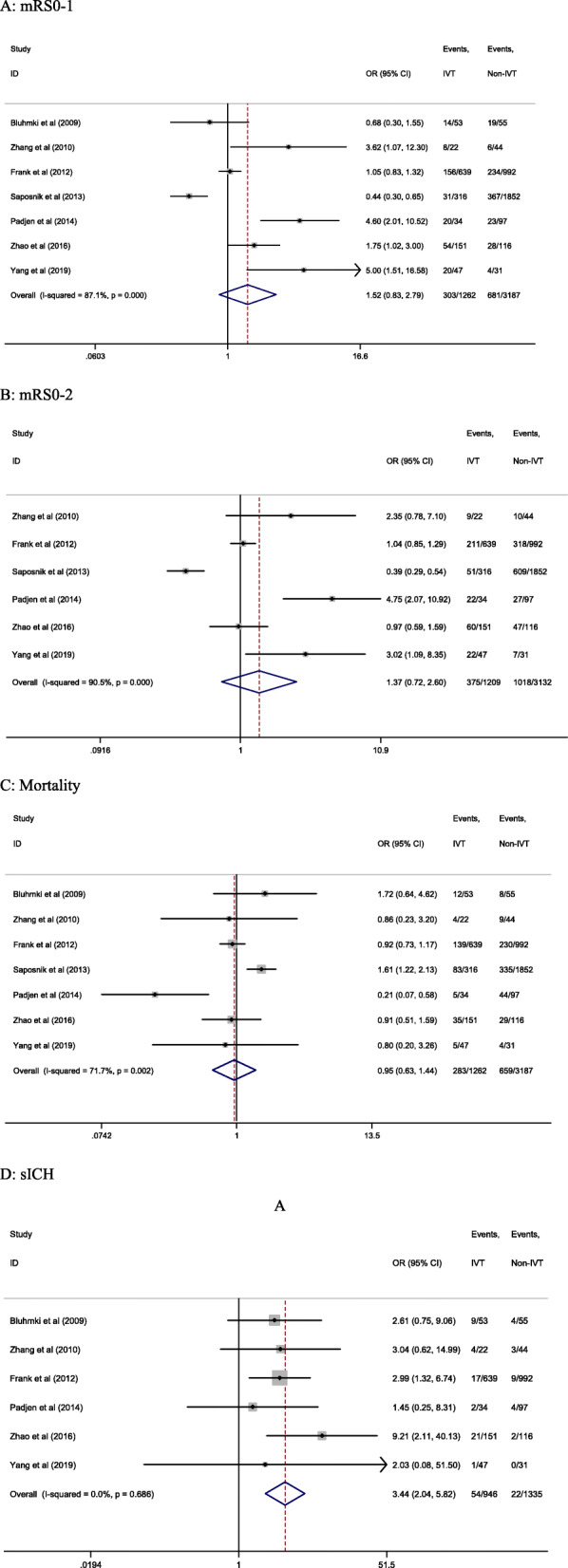
Forrest plot of meta-analysis in AF IVT and AF non-IVT patients. Each study is represented by a point estimate of the OR and the accompanying 95% CIs

In patients with AF, there were no significant differences in the proportion of mRS 0–1 (24.0% vs. 21.4%; OR 1.52; 95% CI 0.83–2.79; *I*^2^ = 87.1%; *P* = 0.172, Fig. 3a), mRS 0–2 (31.0% vs. 32.5%; OR 1.37; 95% CI 0.72–2.60; *I*^2^ = 90.5%; *P* = 0.331, Fig. 3b) scores or mortality (22.4% vs. 20.7%; OR 0.95; 95% CI 0.63–1.44; *I*^2^ = 71.7%; *P* = 0.813, Fig. 3c) between those treated or not with IVT. In contrast, the incidence of sICH was significantly higher in patients treated with IVT therapy (5.7% vs. 1.6%; OR 3.44; 95% CI 2.04–5.82; *I*^2^ = 0.0%; *P* < 0.001) (Fig. 3d).

### Subgroup analysis and meta-regression analysis

Subgroup analysis was performed according to the study design (Table 3). In both prospective and retrospective AF IVT versus non-AF IVT studies, the functional outcome of patients with AF treated with IVT was worse (*P* < 0.001) than in patients without AF, and the mortality (*P* < 0.001) and sICH incidence (*P* < 0.01) were also higher. On the other hand, in AF IVT versus AF non-IVT prospective studies, the results suggested a poorer functional prognosis (*P* < 0.01) and higher mortality (*P* < 0.05) in AF patients treated with thrombolytic therapy than in those who did not receive it, and in retrospective studies, there was a higher sICH incidence in AF IVT patients (*P* < 0.001).

**Table 3:** Subgroup analyses of efficacy and safety of thrombolysis for acute IS with AF based on study design

Study design	mRS 0–1		Test of association		Heterogeneity	
	AF	Non-AF	OR (95% CI)	***P***	***I***^**2**^ (%)	***P***
Retrospective(*n* = 6)	250/886	990/2777	0.68 (0.57–0.81)	< 0.001	75.0	0.006
Prospective(*n* = 4)	101/568	782/2358	0.41 (0.32–0.52)	< 0.001	0.0	0.343
	**IVT**	**Non-IVT**	**OR (95% CI)**	***P***	***I***^**2**^ **(%)**	***P***
Retrospective(n = 4)	238/859	272/1183	1.24 (1.01–1.52)	0.039	74.5	0.008
Prospective(*n* = 3)	65/403	409/2004	0.65 (0.48–0.87)	0.005	92.1	< 0.001
	**mRS 0–2**		**Test of association**		**Heterogeneity**	
	**AF**	**Non-AF**	**OR (95% CI)**	***P***	***I***^**2**^ **(%)**	***P***
Retrospective(*n* = 8)	363/976	1523/2962	0.53 (0.46–0.62)	< 0.001	63.2	0.008
Prospective(n = 3)	138/515	848/1993	0.44 (0.35–0.55)	< 0.001	0	0.474
	**IVT**	**Non-IVT**	**OR (95% CI)**	***P***	***I***^**2**^ **(%)**	***P***
Retrospective(n = 4)	302/859	382/1183	1.10 (0.91–1.33)	0.317	50.6	0.108
Prospective(*n* = 2)	73/350	636/1949	0.53 (0.40–0.70)	< 0.001	96.7	< 0.001
	**Mortality**		**Test of association**		**Heterogeneity**	
	**AF**	**Non-AF**	**OR (95% CI)**	***P***	***I***^**2**^ **(%)**	***P***
Retrospective(n = 8)	204/1044	378/3047	1.86 (1.54–2.25)	< 0.001	49.7	0.053
Prospective(n = 6)	189/983	329/3089	2.30 (1.88–2.82)	< 0.001	25.7	0.242
	**IVT**	**Non-IVT**	**OR (95% CI)**	***P***	***I***^**2**^ **(%)**	***P***
Retrospective(n = 4)	183/859	272/1183	0.91 (0.74–1.13)	0.413	0.0	0.998
Prospective(n = 3)	100/403	387/2004	1.34 (1.04–1.73)	0.025	86.3	0.001
	**sICH**		**Test of association**		**Heterogeneity**	
	**AF**	**Non-AF**	**OR (95% CI)**	***P***	***I***^**2**^ **(%)**	***P***
Retrospective(n = 8)	48/1026	71/3022	1.67 (1.14–2.46)	0.009	0	0.490
Prospective(*n* = 5)	78/939	178/3048	1.56 (1.17–2.07)	0.002	0	0.509
	**IVT**	**Non-IVT**	**OR (95% CI)**	***P***	***I***^**2**^ **(%)**	***P***
Retrospective(n = 4)	43/857	14/1183	4.04 (2.16–7.55)	< 0.001	0.0	0.566
Prospective(n = 2)	11/87	8/152	2.18 (0.80–5.88)	0.126	0.0	0.592

The meta-regression analysis explained the variability between studies based on the mRS 0–1 in is the thrombolysis in AF versus non-AF patients. The results showed as the following: the different online year of studies 55.75%, type of study design 59.01%, and multivariable adjusted R^2^ was 84.37% (Table 4).

**Table 4:** Meta-regression according to methodological covariates of AF patients treated or not with IVT

mRS0–1				
Variable	Number of estimates	Tau 2	R^2^	*P*-value
Year	10	0.1322	55.75%	0.037
Study design	10	0.1367	59.01%	0.033
Stroke severity	8	0.3839	–	0.848
Thrombolysis time window	8	0.5316	–	0.975
Multivariable adjusted R^2^ of the models 84.37%				

*Tau2* variance residual variation between-study due to heterogeneity, *R*^*2*^ Adjuested R^2^ of the Meta-regression model

### Assessment of quality and publication bias

**Fig. 4:**
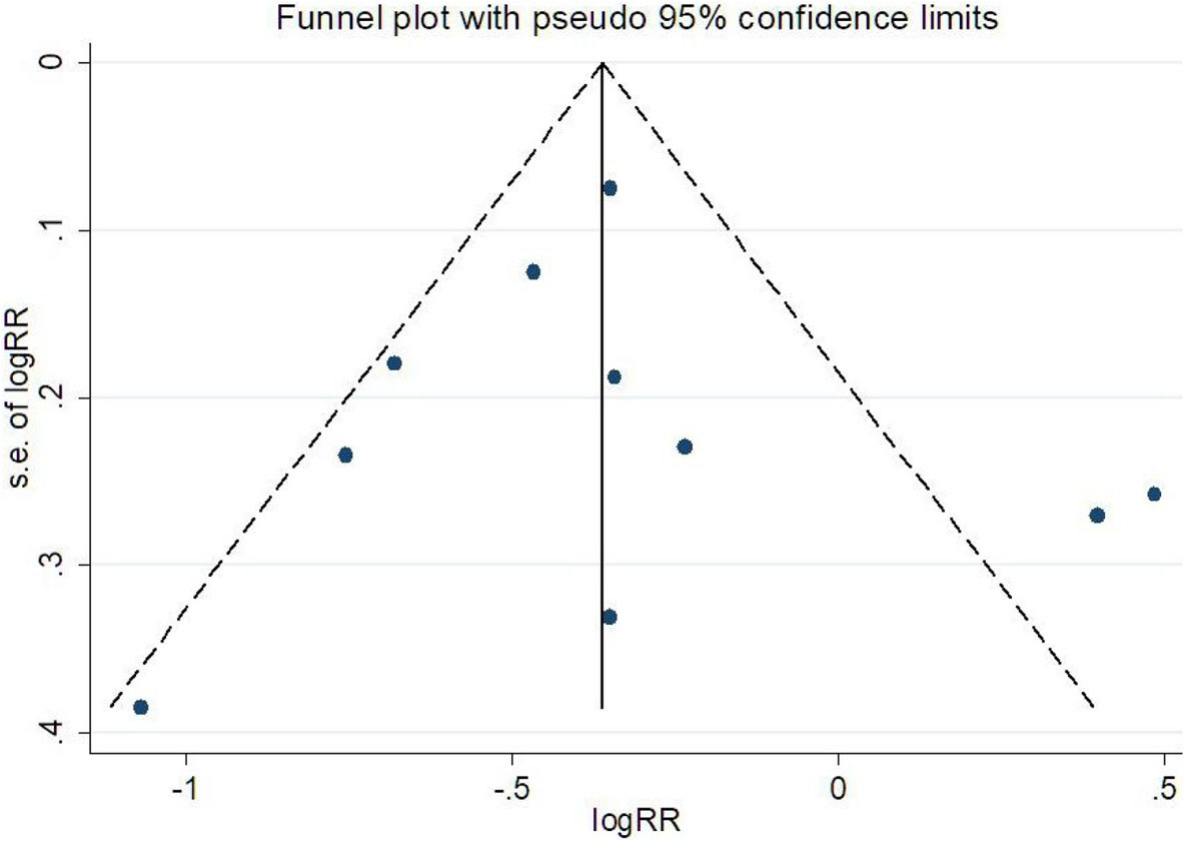
Funnel plot of publication bias in the meta-analysis. The largest studies are plotted near the average, and smaller studies are spread evenly on both sides of the average, creating a roughly funnel-shaped distribution. Deviation from this shape can indicate publication bias

Most included cohort studies were of high quality, with NOS scores ranging from 6 to 9 (Table 5). The mean NOS for all included studies was 7 and the RCT (Bluhmki et al. 2009) assessed with the Cochrane collaboration tool showed a low risk of bias. The study was conducted blindly, with random sequence generation and allocation concealment. The outcome data in the included RCT study was subjective. A funnel plot and Egger’s test were performed to evaluate publication bias in this meta-analysis. Egger’s test (*P* = 0.783) showed there was no significant evidence of publication bias. On the other hand, no significant publication bias was detected based on Begg’s funnel plot (Fig. 4).

**Table 5:** Quality assessments of the included studies with the NOS

Study	Selection	Comparability	Outcome	Total score
Mehrpour et al. 2019 [13]	****	*	**	7
Yang et al. 2019 [15]	****	*	***	8
Zhao et al. 2017 [12]	***	**	**	7
Zhao et al. 2016 [21]	****	**	**	8
Tu et al. 2015 [19]	***	**	**	7
Al-khaled et al. 2014 [17]	***	*	*	5
Saarinen et al. 2014 [18]	***	**	***	8
Padjen et al. 2014 [20]	***	**	**	7
Padjen et la 2013 [10]	****	*	***	8
Saposnik et al. 2013 [11]	****	*	*	6
Sung et al. 2013 [14]	***	**	**	7
Frank et al. 2012 [9]	***	*	**	6
Seet et al. 2011 [8]	***	**	**	7
Awadh et al. 2010 [25]	****	*	**	7
Sanak et al. 2010 [16]	****	**	*	7
Zhang et al. 2010 [7]	***	**	**	7
Bluhmki et al. 2009 [5]	****	**	***	9
Kimura et al. 2009 [6]	***	*	**	6

## Discussion

Patients with stroke and AF have poorer neurological outcomes than those without AF [25]. AF appears to be an independent risk factor for in-hospital mortality, length of hospital stay, and increased treatment costs in stroke patients [26]. Kimura et al. reported that AF was independently associated with no early recanalization after IVT in acute IS [27]. A meta-analysis done by Yue et al. in 2016 showed that AF was associated with poor outcomes in thrombolyzed patients with acute IS [28]. With increasing number of studies reporting on the effects of thrombolysis in AF patients, we collected more evidence to compare the outcome of thrombolytic therapy in acute IS patients with or without AF, as well as of thrombolytic versus nonthrombolytic therapy in patients with AF. This allowed us to reach more comprehensive conclusions regarding the safety and efficacy of thrombolysis in acute IS patients with AF.

The final meta-analysis included 18 studies. Sixteen studies compared thrombolytic outcomes in acute IS patients with or without AF. These studies included 8509 patients (24.97% of them with AF). Seven studies compared the outcome in AF patients treated or not with IVT. These studies included 4449 patients, 28.36% of whom received thrombolytic therapy. Five studies included both comparisons. Comparison of AF IVT versus non-AF IVT groups showed that AF was associated with a significantly lower proportion of patients with mRS of 0–1, mRS of 0–2 90 days after stroke and significantly higher mortality and sICH incidence. Comparison of AF IVT versus AF non-IVT groups showed that thrombolysis carried a higher risk of sICH and was not associated with a better prognosis. Subgroup analysis in prospective studies also suggested a poorer functional prognosis and higher mortality in AF patients treated with IVT when compared with those who did not receive IVT.

The explanation for the observed results are as follows: First, patients with AF have greater infarct sizes and worse collateral circulation, resulting in worse baseline symptoms [15, 16]. Second, patients with AF appear to be more likely to have large or old thrombi, which are resistant to IVT [6]. In most of the included studies, baseline NIHSS was higher in the AF group than in the non-AF group. In addition, some AF patients were treated with anticoagulant therapy prior to IVT, which also increases the risk of bleeding. A meta-analysis showed that the risk of sICH after thrombolytic therapy was higher in patients receiving warfarin with subtherapeutic INR levels [29]. How to improve the efficacy and safety of thrombolysis in patients with AF is worthy of further studies. New thrombolytic drugs [30], tele-thrombolysis [31] and combination with mechanical thrombectomy [32] may offer new options.

Heterogeneity was present in several statistical results of our study. Potential confounders between the groups were not balanced during comparative analysis, which may be a source of heterogeneity. In the included studies, patients with acute IS and AF were generally older and had a higher NIHSS, but we did not adjust for age or NIHSS in the meta-analysis. For example, in the comparative analysis of mRS 0–1 proportions between the AF-IVT and AF non-IVT groups, the *I*^2^ was 71.0%. Based on sensitivity analysis, heterogeneity was derived from two studies [14, 15]. The causes of heterogeneity in these two studies were analyzed: one study [14] included more severe stroke (NIHSS> 10) patients, while the other study [15] included patients with a longer thrombolysis time window (3-9 h) than the others. Excluding these two studies did not change the statistical results, but the heterogeneity improved greatly (OR 0.52; 95% CI 0.45–0.60; *I*^2^ = 30.6%; *P* < 0.001). In addition, the specific type of AF also explained the heterogeneity. Seet et al. reported that patients with chronic AF had worse outcomes than non-AF patients, and was greater in patients with AF of longer duration [8]. Meanwhile the meta-regression analysis showed that the heterogeneity may related to the different online year of studies and type of study design.

We acknowledge that our study has several limitations. First, there was only one RCT study (out of 18 studies). Second, sample sizes in most studies were relatively small and varied between groups, which may limit the analytical capacity. Third, in the included studies, the IVT time window was not uniform: in some cases it was less than 3 h, in others less than 4.5 h, and in one case 3–9 h. Fourth, whether intravenous thrombolysis was combined with mechanical thrombectomy was not mentioned in the included studies. Finally, individual data were insufficiently detailed to identify subgroups according to age range, baseline NIHSS score, onset to needle, type of AF and other clinical factors that may influence the efficacy and safety of thrombolysis in AF patients. Further studies with a double-blind design, larger sample sizes and well-matched patient characteristics should be considered.

## Conclusions

Acute IS patients with AF had worse outcomes than those without AF after thrombolytic therapy as well as a higher incidence of sICH after thrombolysis than those without thrombolysis. Thrombolysis in IS patients with AF should be carefully considered based on clinical factors such as NIHSS score, age, whether patients are taking anticoagulant drugs and type of AF.

## Data Availability

All data generated or analyzed during this study are included in this publication.

## Abbreviations

IS: Ischemic stroke
AF: Atrial fibrillation
IVT: Intravenous thrombolysis
mRS: Modified Rankin Scale
sICH: symptomatic intracranial hemorrhage
